# Arthroscopic Augmentation of the Anterior Capsule Using Artificial Tape for Borderline Developmental Dysplasia of the Hip

**DOI:** 10.1016/j.eats.2025.103534

**Published:** 2025-04-03

**Authors:** Jin Zhang, Shi-yu Sha, Tao Liang, Yi Liu, Qing-Feng Yin

**Affiliations:** a Department of Sports Medicine, Beijing Jishuitan Hospital, Capital Medical University, Beijing, China; b Department of Sports Medicine, The Second Hospital of Shandong University, Jinan, China; c Department of Orthopedics, The First People‘s Hospital of Ningyang County, Taian, China

## Abstract

With the recognition of the positive effect of the iliofemoral ligament in maintaining hip stability, particularly in patients with borderline developmental dysplasia of the hip, some surgeons recommend restoring its native anatomy through conservative capsulotomy and final closure. In the many different kinds of capsulotomy, longitudinal capsulotomy is a technique that can reduce damage to the iliofemoral ligament. This protective effect is sufficient to restore hip stability in patients with femoroacetabular impingement but may be inadequate for patients with borderline developmental dysplasia of the hip because of the laxity and capsular instability. Therefore, we propose a surgical technique that utilizes artificial tape to augment the anterior capsule under longitudinal capsulotomy.

Hip arthroscopy is an effective and innovative procedure with rapidly expanding indications.^1^ It has achieved positive outcomes in managing femoroacetabular impingement and is gradually being applied to patients with borderline developmental dysplasia (BDDH). The utility of the capsulotomy in hip arthroscopy is well known. In the previous reports, the interportal capsulotomy has been the primary procedure employed to access the hip joint, involving a transverse incision in the capsule. However, the transverse incision may lead to potential hip instability and adverse clinical outcomes, especially in patients with BDDH. The incision of the longitudinal capsulotomy is made along the neck of the femur, causing less damage to the iliofemoral ligament than the transverse incision, which helps preserve the integrity of the capsule and provides mechanical advantages. However, the instability in patients with BDDH is related to not only damage of the iliofemoral ligament but also thinning of the anterior hip capsule,^2^ and just protecting the iliofemoral ligament may not suffice to uphold hip stability. Therefore, alternative approaches should be considered to enhance hip stability. In this report, we introduce a technique for anterior capsule mechanical augmentation with artificial tape based on the longitudinal capsulotomy.

### Preoperative Evaluation and Indications

The preoperative evaluation should include general health, current medical history, and trauma history. A thorough preoperative physical examination should assess hip mobility and laxity of the joint, as well as specific tests such as range of motion, the Anterior Hip Apprehension Test, the Flexion Adduction External Rotation Test, the Flexion Abduction External Rotation Test, and Beighton score. A comprehensive radiographic evaluation encompasses an anteroposterior view of the pelvis, Dunn’s view, and the false profile view to assess acetabular coverage. Additionally, a magnetic resonance imaging scan helps to determine the labrum and ligament injuries and the character of the capsule.

Patients with BDDH who have a positive test of anterior apprehension with anterior microinstability or anterior capsular thinning are the primary candidates for this technique.

## Surgical Technique

### Anesthesia and Positioning

The surgical procedure is conducted under general anesthesia. The patient is placed in the supine position on a traction table with the operative limb positioned in a neutral position of abduction-adduction with 5° to 10° of flexion while the contralateral side is placed in an abduction position of 45°.

### Establishment of Portals and Capsulotomy

The surgery is performed through standard anterolateral, mid-anterior, and distal anterolateral accessory portals. The longitudinal “outside-in” capsulotomy technique is utilized for a capsular incision, which is made roughly perpendicular to the indirect head and is extended to the anterior inferior iliac spine proximally below the indirect head. Partial release of the subspinous attachments of the capsule is performed to facilitate the acetabular trim and labrum repair. The distal end of the incision is extended to the zona orbicularis to ensure adequate management of the cam lesion at the head-neck junction. Intra-articular management is performed as routine.

### Capsule Closure Reinforced With Artificial Ligaments

**Fig 1:**
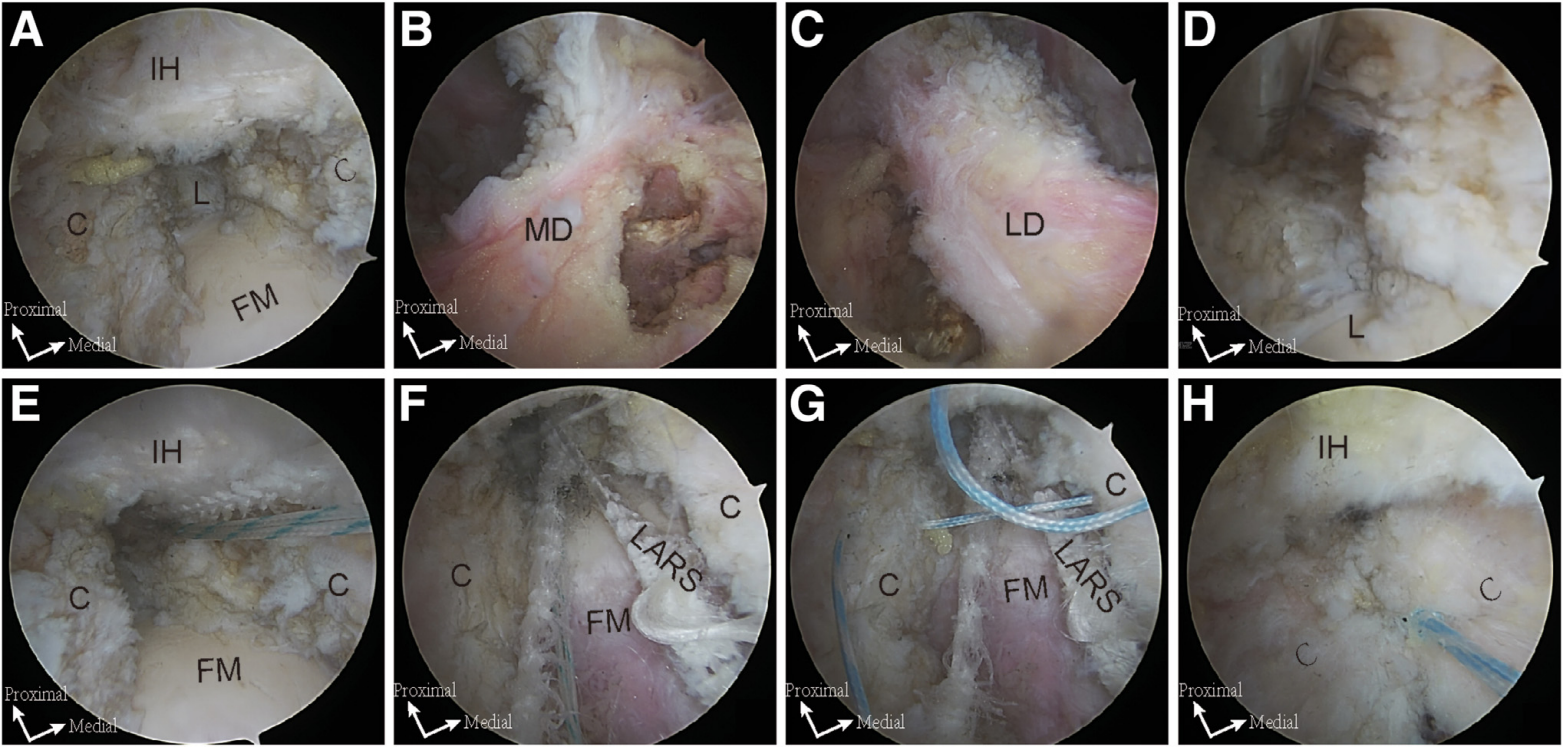
Arthroscopic views of the right hip show the key steps of the augmentation procedure with a 30° scope. The patient is positioned supine on a traction table, with the operative limb in neutral rotation and slight flexion. (A) The longitudinal capsular incision is extended proximally to the labrum and distally to the femoral neck through the mid-anterior portal. (B, C) The fixation points of the medial and lateral bundles of the iliofemoral ligaments are identified and marked with a radiofrequency probe. (D) A proximal tunnel is predrilled. (E) An anchor loaded with LARS suture tape is placed in the proximal tunnel through the distal anterolateral accessory portal. (F) The LARS is fixed and tightened in the extension position. (G) Sutures are passed through each side of the incised capsule. (H) Final arthroscopic view showing complete capsular closure. (C, capsule; FM, femoral head; IH, indirect head; L, labrum; LARS, Ligament Advanced Reinforcement System; LD, lateral bundles of iliofemoral ligament; MD, medial bundles of iliofemoral ligament.)

**Fig 2:**
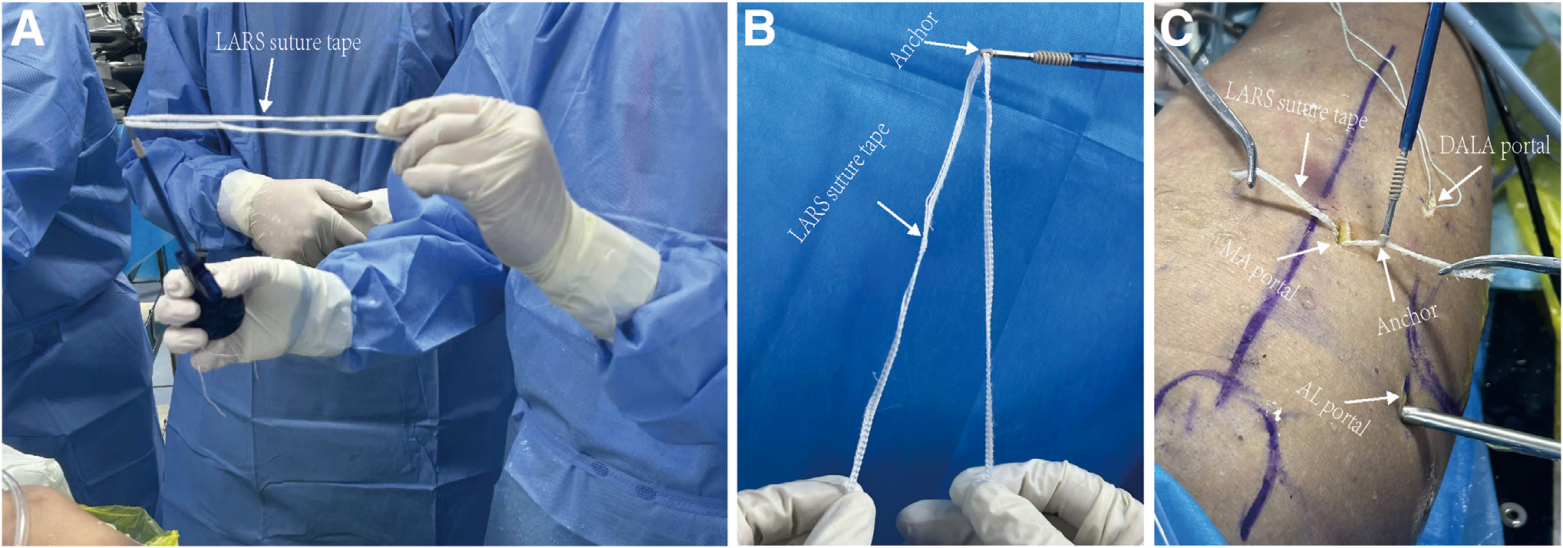
Preparation of the Ligament Advanced Reinforcement System suture tape. (A, B) The Ligament Advanced Reinforcement System suture tape is passed through the hole of the anchor to create a fold. (C) After the first anchor is placed in the proximal tunnel, the ends of the tape are retrieved from the mid-anterior portal and passed through the hole of the second and third anchors.

**Fig 3:**
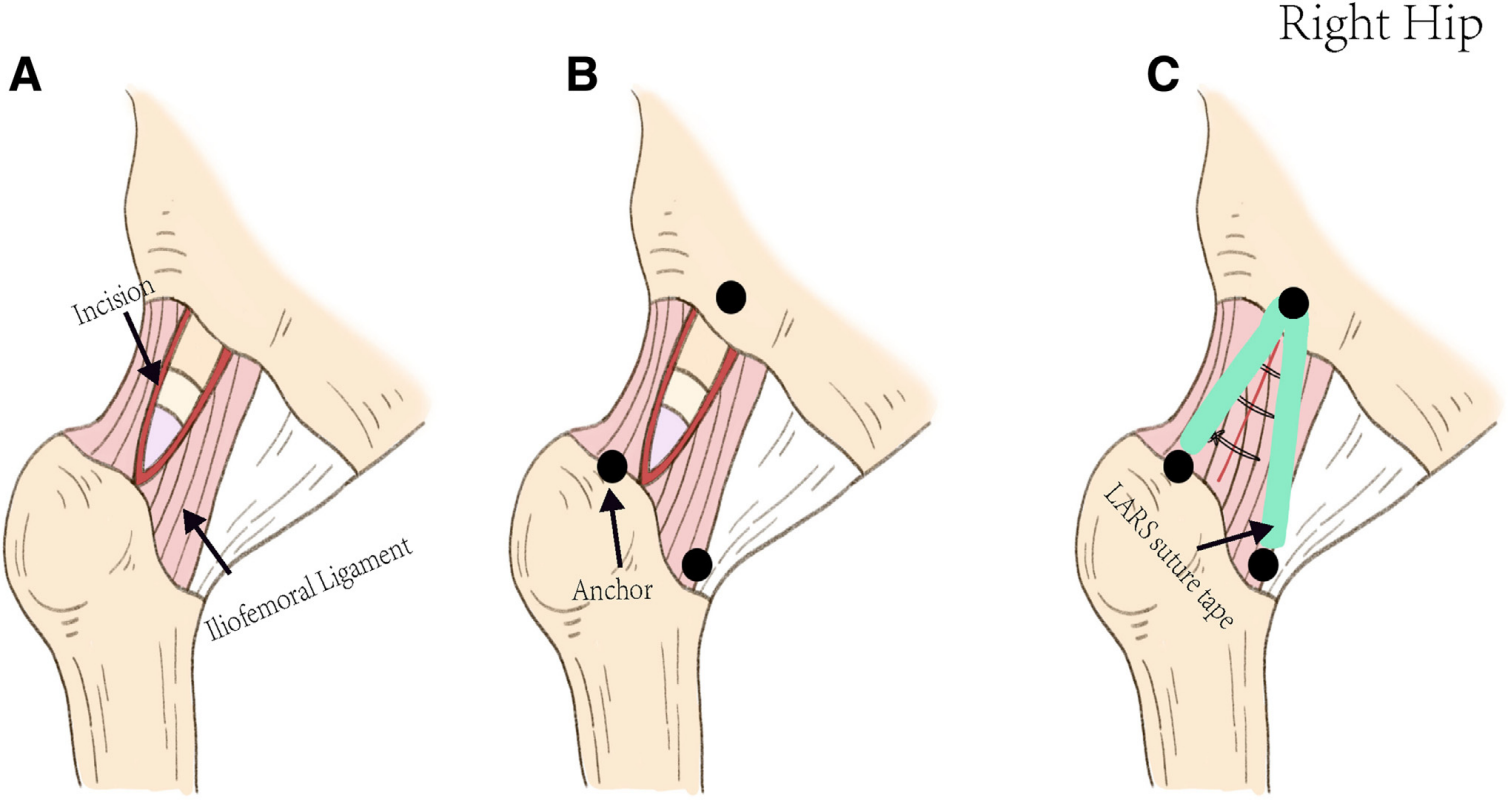
The schematic diagram of the right hip shows the main procedures of the augmentation technique from the mid-anterior portal. (A) The longitudinal “outside-in” capsulotomy technique is performed for the capsular incision. (B) Fixation points are identified: subspine region, the end of the lateral bundles, and medial bundles of the iliofemoral ligament. (C) The Ligament Advanced Reinforcement System suture tape is fixed to the 3 fixation points to augment the iliofemoral ligament, followed by side-to-side suturing of the longitudinal incision.

The localization of the fixation points for the artificial ligament is first conducted before the closure of the hip capsule. The proximal fixation point is selected in the subspinal region, 5 mm above the superior margin of the labrum; the distal lateral point is located at the end of the lateral bundles of the iliofemoral ligament (the outer third of the intertrochanteric line), and the distal medial point is located at the end of the medial bundles of the iliofemoral ligament (intertrochanteric line). These points are marked with a radiofrequency probe (Fig 1A-C). After that, the tunnels are predrilled with a 4.5-mm anchor awl (Fig 1D). A 3-mm LARS suture tape (LARS AC; LARS) is passed through the hole of a 5.5-mm GripLoc PEEK (polyether ether ketone) Knotless Anchor (Allvacon Orthopedics) to create a fold (Fig 2 A and B). The GripLoc loaded with LARS suture tape is placed into the proximal tunnel through the distal anterolateral accessory portal (Fig 1E). Then, the ends of the suture tape are retrieved from the mid-anterior portal and passed through the hole of the second and third GripLoc (Fig 2C). Finally, both anchors are placed into the distal medial and lateral bundles, respectively. The ligament is fixed and tightened in the extension position with the longitudinal capsule incision sutured by the No. 2 Orthocord suture (DePuy Mitek) in a side-to-side fashion (Fig 2F-H). Figure 3 is the schematic diagram of the main procedures. For the detailed procedure, refer to Video 1.

### Postoperative Rehabilitation

Routine postoperative analgesia is provided, along with a 4-week course of medication to prevent heterotopic ossification. Excessive external rotation and hip extension within the first 6 weeks postoperatively should be avoided. Weightbearing is restricted with crutches for 4 weeks.

## Discussion

The joint capsule is essential for maintaining hip joint stability. It is composed of 4 ligaments, with the iliofemoral ligament being the strongest anteriorly. The interportal capsulotomy is the most commonly used technique in hip arthroscopy, offering sufficient visualization and full instrument mobility. However, the transverse incision may lead to joint instability. Khair et al.^3^ reported in a cadaveric study that increasing the size of the interportal capsulotomy linearly decreased the force required to distract the hip. The incision of the longitudinal capsulotomy is proximally aligned with the direction of the iliofemoral ligament, minimizing the damage to the ligament. Routine side-to-side sutures can basically restore the original anatomy of the iliofemoral ligament. Yin et al.^4^ have evaluated the clinical outcomes of the longitudinal capsulotomy and demonstrated its security, feasibility, and clinical efficacy. However, the anterior hip capsule is thinner in patients with BDDH,^5^ and simply performing the longitudinal capsulotomy may not be sufficient to maintain hip stability. Therefore, it is necessary to explore alternative methods to further strengthen the iliofemoral ligament.

The Ligament Advanced Reinforcement System (LARS), composed of synthetic polyethylene terephthalate, has attracted much attention in sports medicine because of its high tensile strength, fatigue resistance, and stiffness. LARS is widely used for ligament augmentation in the shoulder, knee, and ankle, with limited reports in the hip.^6^^,^^7^ Although LARS has not been applied in hip arthroscopy, its successful use in other joints suggests its feasibility for mechanical substitution of the iliofemoral ligament in hip arthroscopy. Given the challenges of shaping and manipulating the “mesh” variant of the LARS, we choose the “tape” LARS to mimic the function of the iliofemoral ligament, positioning it in triangular or inverted “Y” configurations based on the ligament’s anatomy. This technique does have disadvantages, including excessive ligament tension and restricted joint mobility, which require further investigation and refinement. Table 1 details the advantages and disadvantages of our technique. Pearls and pitfalls of our technique are detailed in Table 2.

**Table 1:** Advantages and Disadvantages of Augmentation of Iliofemoral Ligament With the Ligament Advanced Reinforcement System

Advantages	Disadvantages
• Reinforcing the iliofemoral ligament anatomically in conjunction with joint capsule suturing provides mechanical stability for patients with BDDH	• High additional costs
• Performed entirely under arthroscopy with no need for additional approaches or incisions, resulting in minimal injury	• Difficult arthroscopic technique
• The procedure is straightforward	• Additional anchors and sutures may be required

BDDH, borderline developmental dysplasia of the hip.

**Table 2:** Pearls and Pitfalls of Augmentation of Iliofemoral Ligament With the Ligament Advanced Reinforcement System

**Pearls**
• Preservation of the vertically incised joint capsule
• Positioning of the additional tape fixation points
• The tension of the tape fixation should not be too tight
• Protection and restrictions during early postoperative rehabilitation
**Pitfalls**
• Joint capsule tissue is difficult to preserve
• Inaccurately positioned the tape fixation points
• High tension of the tape fixation

In summary, using LARS to augment the iliofemoral ligament provides a feasible and effective method for capsule augmentation in patients with BDDH.

## Disclosures

All authors (J.Z., S-y.S., T.L., Y.L., Q-F.Y.) declare that they have no known competing financial interests or personal relationships that could have appeared to influence the work reported in this paper.

## Supplementary Data

Video 1 The patient is lying supine on a traction table with the right limb positioned in a neutral position. The portals we use are anterolateral, mid-anterior, and distal anterolateral accessory. The longitudinal capsulotomy technique is used for the incision of the capsule through the mid-anterior portal. First, the localization of the fixation points of the Ligament Advanced Reinforcement System (LARS) is conducted. After that, the tunnels are predrilled in the 3 points. Then, the LARS suture tape is passed through the hole of the first anchor to create a fold, and the anchor is placed into the proximal tunnel through the distal anterolateral accessory portal. When the first anchor is placed, the 2 ends of the LARS suture tape are retrieved from the mid-anterior portal and passed through the hole of the second and third anchors, respectively. Finally, the 2 anchors are placed into the distal medial and lateral tunnels. The LARS is fixed and tightened in the extension position with the longitudinal capsule incision sutured in a side-to-side fashion.
